# Event and Comment

**Published:** 1919-10

**Authors:** 


### DENTAL REGISTER

THE MAGAZINE OF DENTISTRY

Vol. LXXIII OCTOBER, 1919 No. 10

### EVENT AND COMMENT

**Is the Present Practice of Dentistry Benefiting Humanity?** We are reprinting in this issue the address of Dr. Eugene S. Talbot as chairman, and after his retirement as secretary for over thirty years, of the Section of Stomatology of the American Medical Association. It is interesting in many ways. It has an historical value because of the contents and comments of the essayist. There is one statement made with characteristic candor and confidence that we are not sure the profession of dentistry will endorse with any considerable degree of enthusiasm, which we quote here for the careful consideration of those who may have, unconsciously perhaps, fallen into the habit of thinking that they had spent their lives in a consistent endeavor to do something worth while for humanity, and are told of their failure in these words: ''Our present method of treatment has not benefited the race as a whole. Decay of the teeth and deformities of the jaws have increased from generation to generation until at the present time hardly a child under 12 years of age can be found who is free from tooth decay and deformities of the teeth and jaws. Since our present methods of procedure are faulty, we must resort to radical changes in treatment and devote our entire attention to preventive measures.'' The statement that ''our present method of treatment has not benefited the race as a whole,'' we are not prepared to endorse, for the reason that there is abundant evidence to convince any thoughtful person who is acquainted with the health conditions of the teeth of the American people as compared with people of any other nation. And the same may be said of the general physical health of our people when compared with the people of other nations who have not had similar technical attention given to their teeth. There is no doubt that dental caries, malposed teeth, and peridental diseases are prevalent, but we can not agree that this is due to the fact that the dental profession has purposely neglected to study pathology in order that greater technical skill should be cultivated, even on the theory that the technical art would best prevent the encroachment of dental diseases. As a matter of fact it has been the universal comment of Europeans that the American soldiers not only excelled in valor but in physical well-being and their teeth have been admired and praised almost as much as their good behavior. Most students of dental education have believed that it was a fortunate day when Harris and Hayden gave up their endeavors to secure recognition from the medical profession and organized a distinct department of the healing art, which has developed into a profession because it was established on the best-known traditions of medical science and art. Eighty years is a short time in which to bring to perfection a system of practice in the healing art, but it will be difficult we imagine to name any other profession that has made relatively greater progress in the same period. It is true that dentistry has had constant access to all the development in medical science of its time and has gladly welcomed every new achievement and adapted every new discovery to the perfection of her own system of healing.

Dentistry has made many mistakes in the practice as well as the training of her students. The system of dental training is to-day far from perfection, and the same may be said of medicine, but the spirit of progress is alive in dentistry to-day, to a degree that has never been known. While the old traditions may be retarding her progress in some degree, there is a strong current of watching and waiting for the appearing of any helpful agency that can be utilized to better her usefulness in the ministry for good health. Can there be a more characteristic example of this purpose than the practical unanimity with which the profession has accepted and acted upon the discovery of the pathologic relations of diseased teeth to general systemic health? Medical science had only to call attention to the fact and dental practitioners as well as colleges of instruction with earnest enthusiasm set about making the correction. Dr. Talbot in the last sentence of the paragraph quoted above, says: ''Since our present methods of procedure are faulty, we must resort to radical changes in treatment and devote our entire attention to preventive measures.'' It is practically and almost universally believed to-day that the acme of technical attainment has not been achieved, but there is a steady devotion to tested treatments which assures for the near future a degree of conservatism in practice that will prevent any radical upheaval that might bring more disaster than benefit. ''Safety first'' is a good catch phrase to keep the profession steady in these times of change. There is great encouragement in the idea of preventive dentistry, and we firmly believe that it will make great achievements in both medicine and dentistry, in fact we shall be disappointed if dentistry does not win out in this particular; but we do not believe preventive dentistry will come by way of a radical upsetting of our present methods of practice or education. Evolution is a much safer and better method than revolution. We sincerely pray for the day when preventive treatment shall displace rescue endeavors, but we are convinced that in the very nature of things as they happen in this world, it will never be completely realized.

